# The spatiotemporal distribution of human brucellosis in mainland China from 2007-2016

**DOI:** 10.1186/s12879-020-4946-7

**Published:** 2020-03-27

**Authors:** Peifeng Liang, Yuan Zhao, Jianhua Zhao, Dongfeng Pan, Zhongqin Guo

**Affiliations:** 1 Department of medical record and statistics, People’s Hospital of Ningxia Hui Autonomous Region, Yinchuan, 750004 China; 2 Department of Epidemiology and Biostatistics, School of Public Health and management, Ningxia Medical University, Yinchuan, 750001 China; 3 Ningxia Center for Diseases Prevention and Control, Yinchuan, 750001 China; 4 Department of Emergency, People’s Hospital of Ningxia Hui Autonomous Region, Yinchuan, 750004 China

**Keywords:** Human brucellosis, Mainland China, Geographic information system, Spatial autocorrelation analysis

## Abstract

**Background:**

Despite the considerable efforts made to address the issue of brucellosis worldwide, its prevalence in dairy products continues to be difficult to estimate and represents a key public health issue around the world today. The aim of the present study was to better understand the epidemiology of this disease in mainland China. We set out to investigate the yearly spatial distribution and possible hotspots of the disease.

**Methods:**

Human brucellosis data from mainland China between 2007 and 2016 were collected from the China Information System for Disease Control and Prevention. A geographic information system ArcGIS10.3 (ESRI, Redlands) was used to identify potential changes in the spatial and temporal distribution of human brucellosis in mainland China during the study period. These distributions were evaluated using three-dimensional trend analysis and spatial autocorrelation analyse. A gravity-center was used to analyse the migration track of human brucellosis.

**Results:**

A total of 399,578 cases of human brucellosis were reported during the 10-year study period. The monthly incidence of brucellosis in China demonstrates clear seasonality. Spring and summer are the peak seasons, while May is the peak month for brucellosis. Three-dimensional trend analysis suggests that brucellosis is on the rise from south to north, and that the epidemic situation in northern China is more severe. Between 2007 and 2016, the overall migration distance of the brucellosis incidence gravity-center was 906.43 km, and the direction was southwest. However, the overall gravity center of brucellosis was still in the northern part of China. In the global autocorrelation analysis, brucellosis in China demonstrated a non-random distribution between 2013 and 2014, with spatial autocorrelation (*Z* > 1.96, *P* < 0.05) and a clustering trend, while no clustering trend was found from 2007 to 2012 or from 2015 to 2016. In the local autocorrelation analysis, a Low-Low cluster phenomenon was found in the south of China in 2013 and 2014.

**Conclusion:**

Human brucellosis remains a widespread challenge, particularly in northern China. The hotspots highlight potential high-risk areas which may require special plans and resources for monitoring and controlling the disease.

## Background

Brucellosis, which is also known as undulant fever, is one of the most common zoonoses in the world [1–4]. It is an infectious disease which can be acute or chronic. It is caused by brucella, which is a natural epidemic disease [5]. Brucellosis is globally distributed. More than 170 countries and regions around the world have recorded brucellosis epidemics, accounting for approximately one-sixth to one-fifth of the world’s population [6]. There are between five and six million patients who are living with brucellosis worldwide, the number of cases reported to the WHO each year is 500,000 [7–10]. The Mediterranean region as well as Asia, Central and South America are the areas with the highest incidences [11–15].

In China, the first incidence of brucellosis occurred for two foreign patients in Shanghai in 1905 and was recorded as Malta fever [16]. However, several patients in China with similar clinical symptoms had been observed in the 10 years before that report. Following the 1905 report, three more cases were reported in Chongqing in 1906 [17]. By the 1950s and 1960s, brucellosis had become more prevalent for Chinese people and animals. Subsequently, from the 1980s to the early 1990s, activities for the prevention and control of brucellosis have gradually been introduced in mainland China, meaning that the brucellosis epidemic had been basically controlled [18]. During this time, the human infection rate was just 3%, the incidence rate was only 0.02/100,000 [16]. However, brucellosis has rebounded since 1993, with sporadic cases increasing at a rate of between 30 and 50%. In 2015, the incidence of human cases reached 4.18/100,000. In the ranking of statutory infectious diseases, it rose from 16th place in 2000 to 6th place in 2014. A strong upward trend such as this is extremely rare among all such reported infectious diseases.

Previous studies have demonstrated that the global epidemiology of brucellosis has changed dramatically over the past decades, particularly in industrialized countries [19]. The spatial distribution characteristics of a brucellosis outbreak also have changed distinctly [20], although whether this change has temporal and spatial covariance requires further investigation.

The aim of our study was to use a spatial distribution model to analyze the spatial distribution characteristics and correlations of human brucellosis in mainland China between 2007 and 2016, and so to provide theoretical support for the prevention and control of human brucellosis in the context of local conditions.

## Methods

### Data source

Human brucellosis cases from 31 provinces (municipalities and autonomous regions) in mainland China between 2007 and 2016 were collected through National Population and Health Science Data Sharing Platform, the Data-center of China Public Health Science. The platform was established in 2004 and is more integrated, effective and reliable than the previous case-reporting system [21]. The copyright of data sharing platform belongs to Chinese center for disease control and prevention (CDC). The data on this platform is publicly available, anyone can download the data from platform after the account application being approved. In mainland China, suspected and confirmed cases of human brucellosis must be reported to local and provincial CDC and then to the Chinese CDC (CCDC). In order to meet the case definition, a diagnosis of human brucellosis must not only be accompanied by clinical signs, but must also be confirmed by a serological test or in isolation, in accordance with the case definition from the WHO [22].

### Statistical analysis

#### Descriptive analysis

Human brucellosis cases which occurred between 2007 and 2016 were collected by month and year, and Excel software was used to draw a time series diagram. Data were sorted and described according to time and region.

#### Three-dimensional trend analysis

Both the spatial distribution and incidence trend of human brucellosis were evaluated and visualized via the three-dimensional trend analysis, utilizing ArcGIS10.3 (ESRI, Redlands) software [23]. The three-dimensional trend analysis uses the least square method to establish the binary polynomial regression model, which excludes both accidental and local variation, and displays the general distribution rule of disease in the study area [24]. The X-axis and Y-axis represent the geometric center of specific study region, while the Z-axis represents the human brucellosis. The higher the Z-axis, the higher the incidence [25]. This means that a point in three-dimensional space (X, Y, Z) represents human brucellosis incidences in a study region. The points in the three-dimensional space are projected onto both the XZ plane and the YZ plane respectively. Following this, an optimal fitting line is created according to the scatter plot, fitting polynomial onto the projection plane, while the curve is used to simulate the spatial trend of the disease [26].

#### Gravity-center migration

A common method for studying the migration of matter and energy in geo-distribution is through the use of the ‘gravity-center’. A ‘geographic gravity-center’ can represent the spatial and temporal distribution characteristics of geographical elements. The moving direction of a gravity-center points to the ‘high density’ element of spatial phenomena, while the distance reflects the spatial difference of the variation range of the geographical elements [27].

##### Gravity-center

Using spatial statistical tools (such as the mean center) to calculate the incidence gravity-center of brucellosis each year, we assumed that a region is composed of several sub-regions. Each sub-region has a particle *P*_*i*_ (*X*_*i*_, *Y*_*i*_) and its attribute value is *M*_*i*_, meaning the center coordinate formula of an attribute is [28]:

$$ X=\frac{\varSigma_{i=1}^n{M}_i{X}_i}{\varSigma_{i=1}^n{M}_i}\kern2.12em Y=\frac{\varSigma_{i=1}^n{M}_i{Y}_i}{\varSigma_{i=1}^n{M}_i} $$

X and Y respectively represent the latitude and longitude of the gravity-center of a region’s attribute, while (X_i_, Y_i_) is the geographic coordinate of the central city of the i-th secondary region and M_i_ is the magnitude of an attribute of the i-th secondary region.

##### Migration track

When a certain attribute value in the study area accounts for a large proportion or a rapid growth rate in the population, due to the unbalanced distribution of the spatial attributes, the regional gravity-center will move to this area. This means that a ‘gravity-center offset’ will occur. The gravity-center deviation demonstrates that the attribute develops rapidly in this direction, while the strength of the overall social activity attribute is enhanced. Through the regional gravity-center migration, we can see the migration track and spatial distribution difference of this region’s attribute, which is calculated as follows:

$$ {D}_{ab}=C\times \sqrt{{\left({X}_a-{X}_b\right)}^2+{\left({Y}_a-{Y}_b\right)}^2} $$

Here, a and b represent different years, *D* represents the distance of the gravity center offset between different years, (X_a_,X_b_) and (Y_a_, Y_b_) represent center coordinates of an attribute of the a and b years, respectively. *C* is a constant, representing the coefficient from the coordinate unit of the earth’s surface to the plane distance; *C* = 111.111.

#### Spatial autocorrelation analysis

Tobler’s first law of geography states that geographic objects or attributes are interrelated in spatial distribution. This means that the degree of interaction between things with a close geographical distance is higher than that of things with a long distance [29]. Spatial autocorrelation in GIS helps understand the degree to which one object is similar to other nearby objects. Moran’s I (Index) measures spatial autocorrelation. Spatial autocorrelation measures how much close objects are in comparison with other close objects. Moran’s I can be classified as positive, negative and no spatial auto-correlation. Positive spatial autocorrelation is when similar values cluster together in a map. Negative spatial autocorrelation is when dissimilar values cluster together in a map. This concept was extended to spatial epidemiology, which can be used to demonstrate the spatial distribution of diseases. Therefore, this analysis has its own advantages for recognizing the spatial distribution characteristics and pattern causes of diseases. Spatial autocorrelation can be divided into global and local spatial autocorrelation.

Global spatial autocorrelation can be used to describe the overall spatial distribution pattern of brucellosis in China [30]. Here, global Moran’s *I* value was used as a measure, which ranges from [− 1,1]. *I* > 0 indicates a positive spatial correlation; *I* < 0 indicates a negative spatial correlation; if *I* value is close to 0, no spatial correlation exists. The larger the absolute value of *I*, the stronger the correlation. When |*Z*| > 1.96, *P* < 0.05 was considered statistically significant and there was spatial autocorrelation, this is written in mathematical notation as:

$$ I=\frac{n\sum \limits_{i=1}^n\sum \limits_{j=1}^n{w}_{ij}\left({x}_i-\overline{x}\right)\left({x}_j-\overline{x}\right)}{\sum \limits_{i=1}^n\sum \limits_{j=1}^n{w}_{ij}\sum \limits_{i=1}^n{\left({x}_i-\overline{x}\right)}^2} $$

*I* is Moran’s *I* value, n refers to the number of spatial elements, *x*_*i*_ and *x*_*j*_ are the observed values at cell i and j, $\overline{x}$ is the mean value of attribute values of all spatial units, while *w*_*ij*_ refers to the spatial weight between elements *i* and *j*. The expression of *w*_*ij*_ is as follows:

$$ {w}_{ij}=\left\{\begin{array}{l}1\ \mathrm{If}\ \mathrm{the}\ i-\mathrm{th}\ \mathrm{and}\ j-\mathrm{th}\ \mathrm{spatial}\ \mathrm{units}\ \mathrm{are}\ \mathrm{adjacent}\\ {}0\ \mathrm{If}\ \mathrm{the}\ i-\mathrm{th}\ \mathrm{and}\ j-\mathrm{th}\ \mathrm{spatial}\ \mathrm{units}\ \mathrm{are}\ \mathrm{not}\ \mathrm{adjacent}\\ {}0\ \mathrm{If}\ \mathrm{the}\ i-\mathrm{th}\ \mathrm{and}\ j-\mathrm{th}\ \mathrm{are}\ \mathrm{equal}\end{array}\right\} $$

In mathematical notation, this is:

$$ {z}_1=\frac{\left(I-E(I)\right)}{\sqrt{Var(I)}} $$

The corresponding variance is:

$$ Var(I)=\frac{1}{S_0^2\left({n}^2-1\right)}\left({n}^2{S}_1-n{S}_2+3{S}_0^2\right)-\frac{1}{{\left(n-1\right)}^2} $$

Among them:

$$ {S}_0={\sum}_{ij}{w}_{ij},{S}_1=2{\sum}_{ij}{w^2}_{ij},{S}_2=4{\sum}_i{w^2}_i,{w}_i={\sum}_j{w}_{ij} $$

$$ E(I)=-1/n-1 $$

Where *E*(*I*) refers to the mean value of spatial Morn’s *I* under the completely random assumption theoretical.

Local spatial autocorrelation: Local cluster analysis was performed using the local Moran’s *I* statistic [31]. Anselin’s local Moran’s *I* (local indicators of spatial association or LISA) statistic indicates the location of local clusters and spatial outliers [32, 33]. There are four cluster types in a local spatial autocorrelation analysis cluster map: High-High, Low-Low, High-Low and Low-High values, which are relative to neighboring districts [34, 35]. High-High and Low-Low clusters indicate that the observed values of an area are similar to those of the surrounding areas. Clusters of areas which have high crude brucellosis incidence rates (High-High or HH) are considered ‘hotspots’, whereas clusters of areas with low crude brucellosis incidence rates (Low-Low or LL) are ‘cold spots’. High-Low and Low-High clusters indicate that the observed values of an area are larger than (or less than) those of the surrounding areas. Clustering and outlier analysis were used to study local autocorrelation, as they can detect correlations between the disease occurrence of the provinces (municipalities and autonomous regions) as well as neighboring provinces (municipalities and autonomous regions) in mainland China and so identify areas where the disease is concentrated. The formula is as follows:

$$ I=\frac{n^2\left({x}_i-\overline{x}\right){\varSigma}_{j=1}^n{w}_{ij}\left({x}_j-x\right)}{\varSigma_{i=1}^n\left({x}_i-\overline{x}\right)2} $$

Here, the letters represent the same meanings as in the global Moran’s *I* coefficients.

## Results

### Epidemic situation of human brucellosis

#### Time distribution

**Fig. 1:**
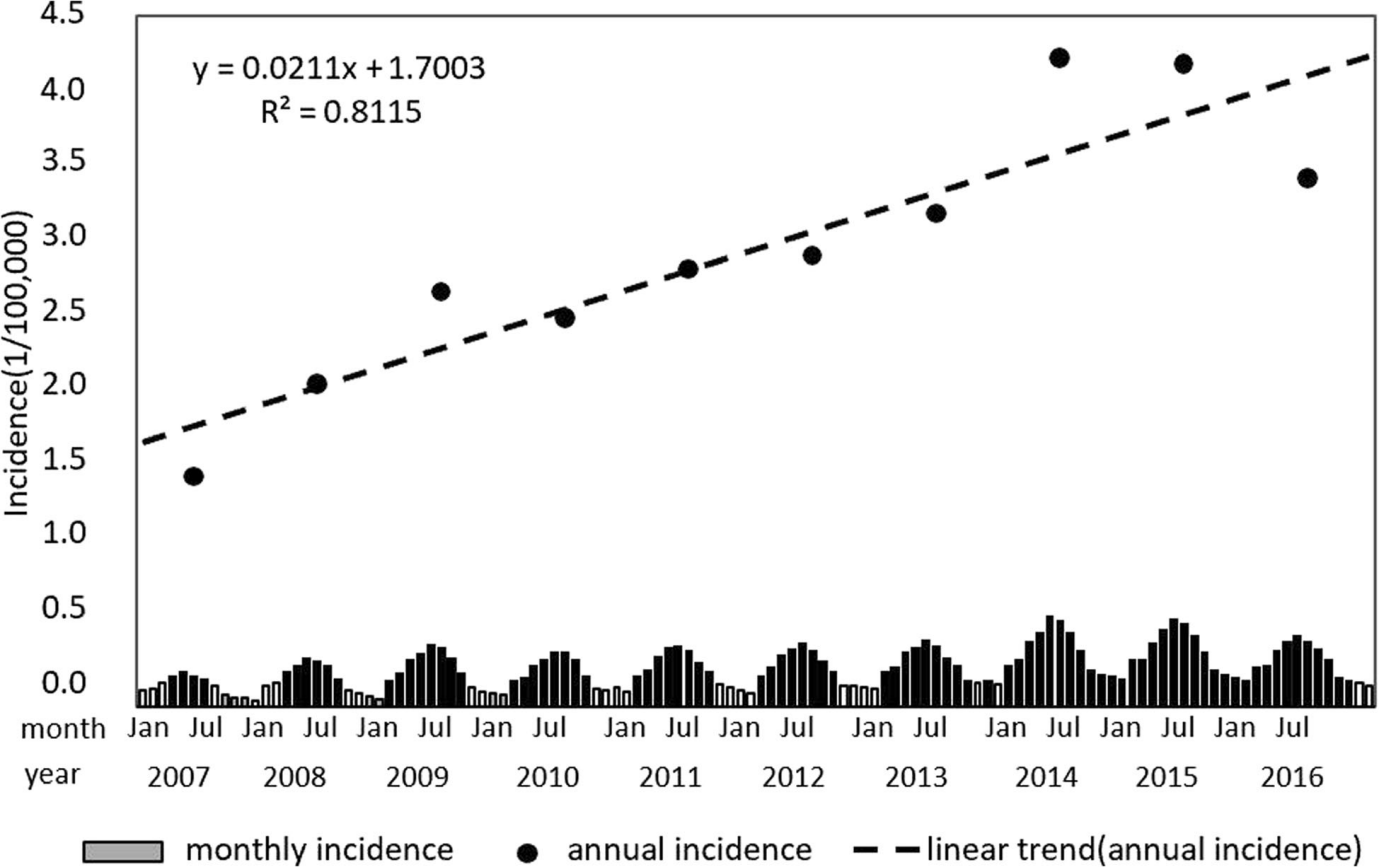
Time distribution of human brucellosis incidences in China from 2007 to 2016. (The histogram shows monthly incidences and the dots show annual incidences of brucellosis. Annual incidences have a y = 0.0211x + 1.7003 relationship. Results of a purely temporal analysis between 2007 and 2016 demonstrated that high-incidence seasons of human brucellosis occurred between late spring and early summer, with incidences highest in May)

During the study period of 2007 to 2016, there were 399,578 reported cases of human brucellosis in mainland China. Annual case totals ranged from 19,721 in 2007 to 57,222 in 2014, with a mean number of approximately 34,498 (95% CI: 25610, 43,386) cases reported per year. The annual mean incidence of brucellosis was 2.97/100,000, with a low of 1.50/100,000 in 2007 and a high of 4.22/100,000 in 2014. As a whole, annual incidences follow a growth trend, with a linear relationship of y = 0.0211x + 1.7003 from 2007 to 2016 (Fig. 1). Furthermore, annual and monthly incidences fluctuated during this 10-year period. Between 2007 and 2009, the annual average incidence of brucellosis in China demonstrated an upward trend. Following that, a short-term decline occurred in 2010, after which incidences of the disease began to rise again, continuing to rise until 2014, and then decline again in 2015 and 2016. A purely temporal analysis between 2007 and 2016 demonstrated that high-incidence seasons of human brucellosis were the time between late spring and early summer, while the incidence was highest in May.

#### Spatial distribution

**Fig. 2:**
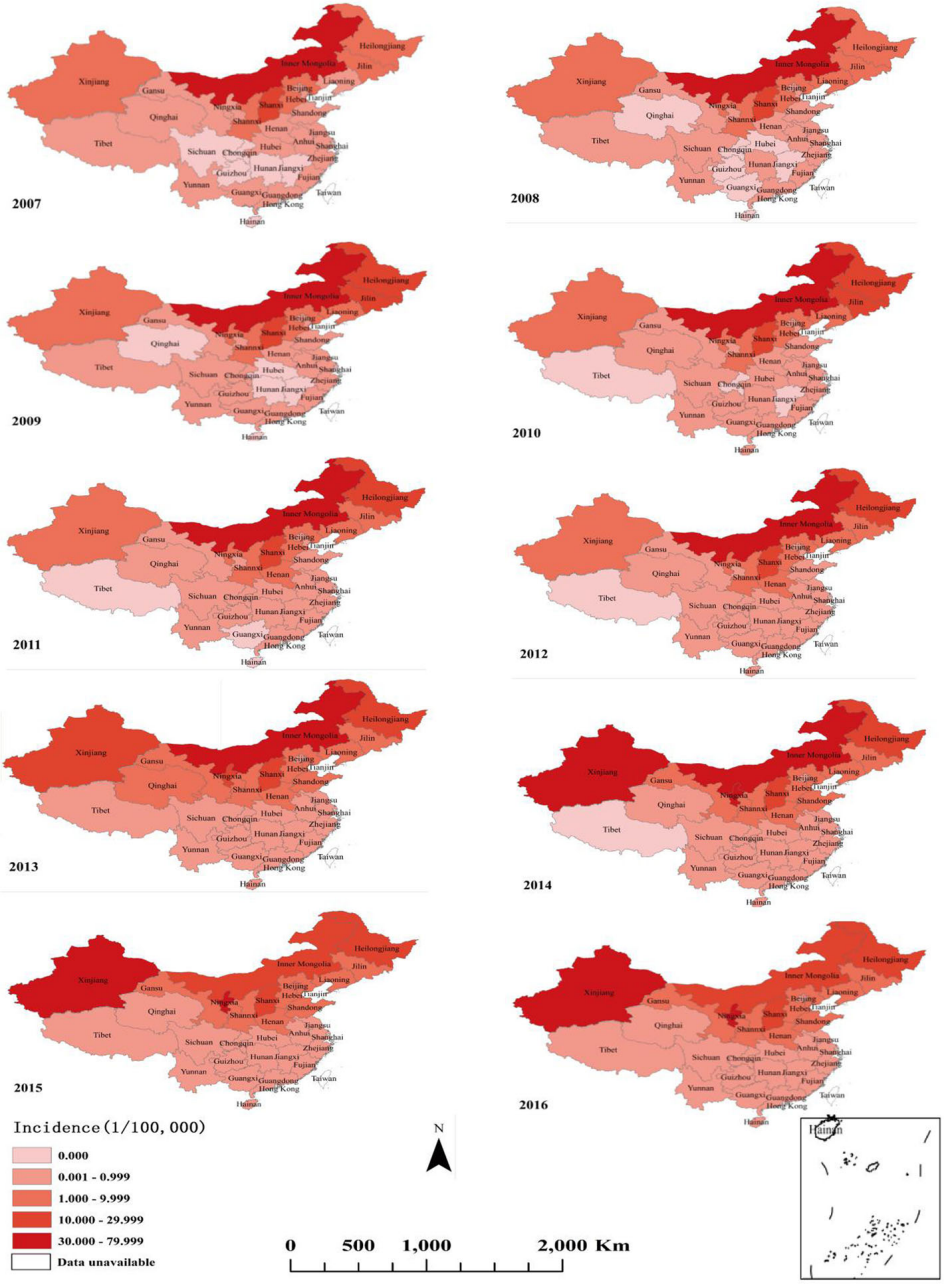
Spatial distribution of human brucellosis incidences in mainland China from 2007 to 2016 (1/100,000). (The map data comes from National Geographic Center of China. After the website application, the data can be downloaded for free. The website link is http://www.ngcc.cn/ngcc/)

Incidences of human brucellosis in each province vary significantly, with an interquartile range of 4.25/100,000 (0.045~4.297/100,000) and a range of 70.10/100,000 (0~70.10/100,000). We defined regions with an incidence greater than 10/100,000 as high incidence areas, while incidences lower than 0.999/100,000 were defined as low incidence areas. Over the total study period, high incidence areas increased from two to five, with the number of high incidence provinces and cities increasing year by year. In 2007, there were six provinces (6/31, 19.35%) with no cases of human brucellosis. These were mainly located in central and southern regions such as Guizhou, Hunan, Jiangxi and Hainan. As time passed, the epidemic area gradually expanded into all provinces and cities in China. The spatial and temporal distribution map (Fig. 2) shows that 31 provinces (municipalities and autonomous regions) had incidences between 2007 and 2016. The epidemic mainly occurred in the north of China, such as the Inner Mongolia and Shanxi provinces. After 2009, the high incidence area grandly expanded in other northern areas, such as the Heilongjiang, Xinjiang and Ningxia provinces. In Inner Mongolia, disease incidence was the highest in history, exceeding the 50/100,000 population between 2009 and 2011. Between 2014 and 2016, incidences increased considerably in areas which had been historically low areas such as Gansu, Qinghai and Shandong. This was most marked in Ningxia, where incidences increased sharply from 0.4636/100,000 in 2007 to 43.6557/100,000 in 2015.

### Three-dimensional trend analysis

**Fig. 3:**
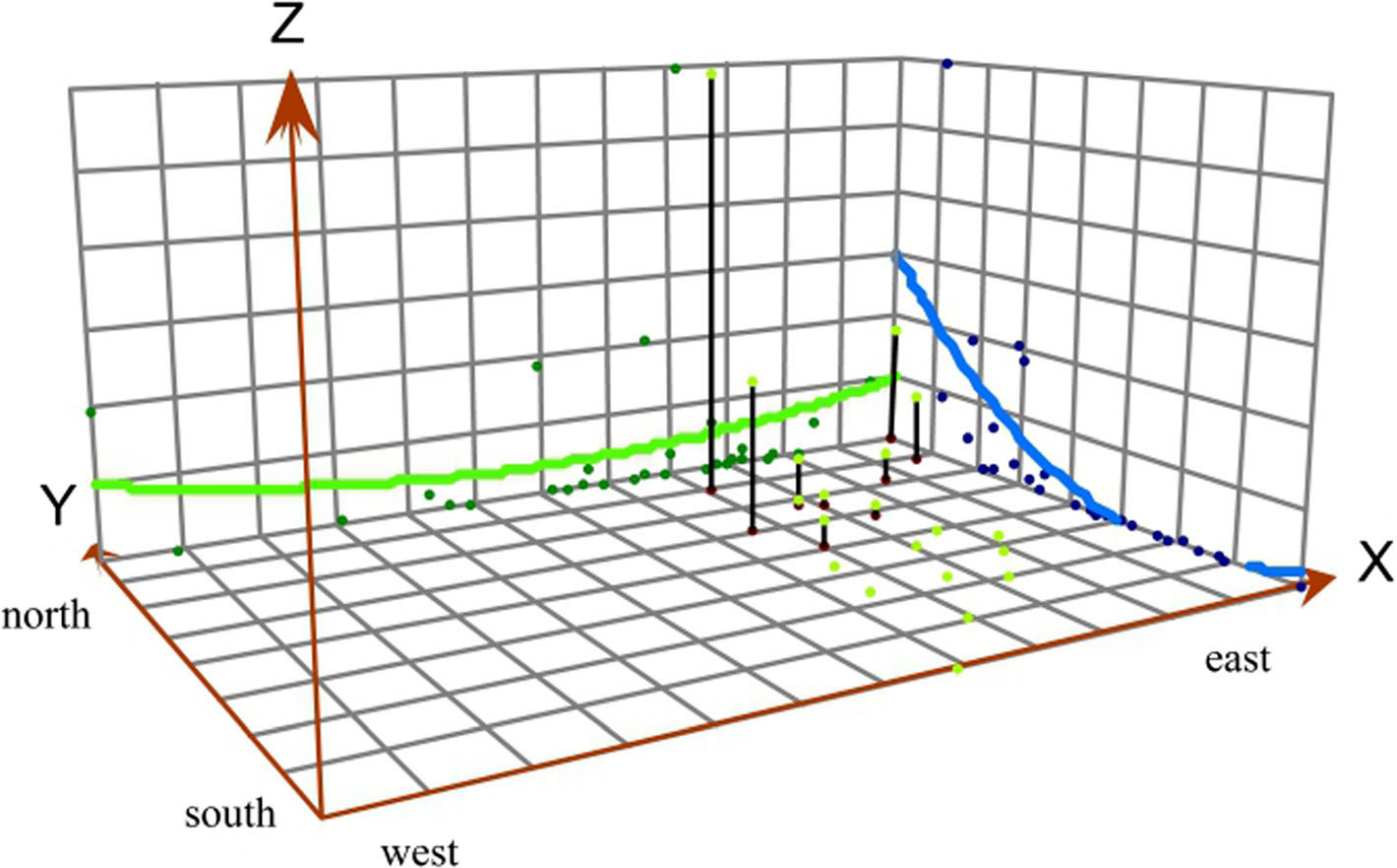
Three-dimensional trend analysis of mean incidences of brucellosis in mainland China between 2007 and 2016. (The curve on the XZ plane represents the East-West direction trend, while the curve on the YZ plane represents change in the North-South direction. Overall, we can see that incidences of brucellosis in northern China are relatively high, although in recent years, incidences in southern China have gradually increased)

The overall three-dimensional trend for 2007–2016 indicated an uneven distribution of human brucellosis in mainland China (Fig. 3). The curve on the XZ plane represents the trend of East-West direction, while the curve on the YZ plane represents the change of North-South direction. Each point represents the projection of incidence. It can be seen that incidences of human brucellosis in the northern region were higher than in the southern region. However, in the east-west direction, incidences of brucellosis were evenly distributed in mainland China.

### Gravity center migration

**Fig. 4:**
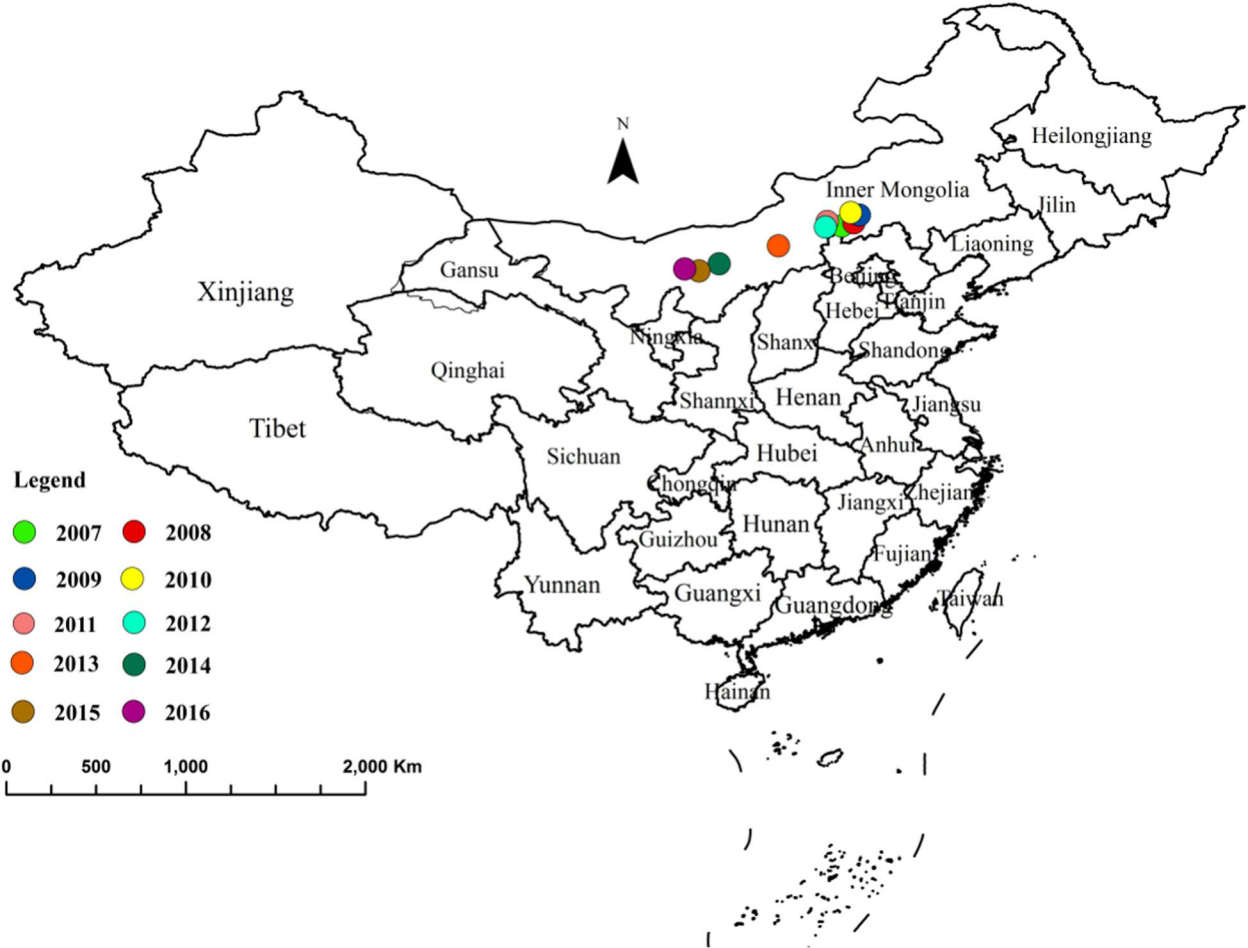
The center of gravity migration track of human brucellosis in China from 2007 to 2016. (The small black dots in the picture show the gravity center of brucellosis between 2007 and 2016. The map data comes from National Geographic Center of China. The website link is http://www.ngcc.cn/ngcc/, Plan approval number is GS (2019)1815)

Most cases of human brucellosis were reported in northern China during 2007 to 2016, while most provinces in northern China experienced a serious epidemic (Fig. 4). Between 2007 and 2012, the gravity-center of brucellosis was concentrated in the central area of Inner Mongolia. After 2012, there was a trend of gradual migration to the southwest. However, the distance and direction of annual migration were not obvious. Results showed that brucellosis was still prevalent in the northern part of China. During the study period, the gravity-center of brucellosis migrated 906.43 km southwest. The distance and direction of annual migration can be seen in Table 1.

**Table 1:** Distance and direction of center of gravity migration of brucellosis between 2007 and 2016

Time	Distance	Direction	Time	Distance	Direction
2007–2008	69.89 km	northeast	2008–2009	53.39 km	northeast
2009–2010	49.95 km	northwest	2010–2011	140.28 km	southwest
2011–2012	31.91 km	southwest	2012–2013	281.90 km	southwest
2013–2014	344.76 km	southwest	2014–2015	119.16 km	southwest
2015–2016	80.72 km	northwest			

### Spatial autocorrelation analysis

#### Global spatial autocorrelation

Using provincial units to carry out the global autocorrelation analysis, we obtained Moran’s *I* value, variance, *Z* score and *P* value from 2007 to 2016, respectively (see Table 2). The values of Moran’s *I* were 0.1179 and 0.1181 respectively for 2013 and 2014, while *Z* values were greater than 1.96 (all *P* < 0.05), indicating that incidences of brucellosis in China between 2013 to 2014 had a non-random distribution, while there was no spatial autocorrelation in either 2007–2012 or 2015–2016 (*Z* < 1.96, *P* > 0.05, meaning there was no clustering trend.

**Table 2:** Global autocorrelation of Moran’s I values of brucellosis in mainland China from 2007 to 2016

Year	Moran’s *I*	Variance	*Z* Score	*P* Value	Aggregation
2007	0.041785	0.002682	1.450621	0.146886	NO
2008	0.051605	0.002669	1.644175	0.100140	NO
2009	0.043654	0.002241	1.626341	0.103877	NO
2010	0.040263	0.002109	1.602407	0.109066	NO
2011	0.043491	0.002197	1.638966	0.101220	NO
2012	0.076004	0.003579	1.827598	0.067610	NO
2013	0.117938	0.004963	2.147162	0.031780	YES
2014	0.118131	0.005379	2.065198	0.038904	YES
2015	0.093595	0.005191	1.761692	0.078121	NO
2016	0.092068	0.005165	1.744833	0.081014	NO

### Local spatial autocorrelation

**Fig. 5:**
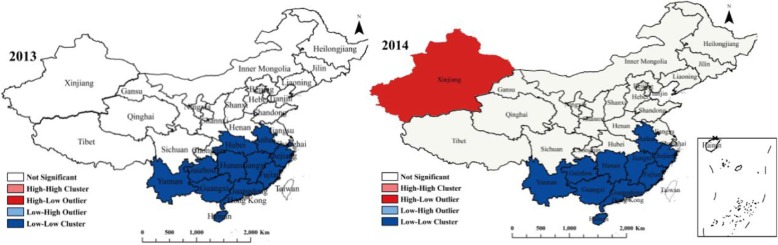
Local spatial autocorrelation analysis of incidences of human brucellosis in mainland China between 2013 and 2014. (Bright red indicates a High-Low association, bright blue indicates Low-High, light red indicates High-High and light blue indicates Low-Low. The map data comes from National Geographic Center of China. The website link is http://www.ngcc.cn/ngcc/)

While global clustering indices evaluate the distribution of events, they do not specify the location of hotspots. To identify areas characterized by a higher rate of human brucellosis incidences, a local spatial autocorrelation analysis was carried out for the years which had a statistical significance of Moran’s *I* value, and a local spatial autocorrelation aggregation distribution map was generated (Fig. 5). There were no High-High, High-Low or Low-High cluster areas in China in 2013, while Low-Low cluster areas tended to be concentrated in the southern provinces of China, including the provinces of Yunnan, Guizhou, Guangxi, Hunan, Guangdong, Hubei, Jiangxi, Fujian, Zhejiang and Anhui, with an average incidence of 0.08/100,000. The new High-Low cluster area in 2014 was Xinjiang, while the Low-Low cluster area decreased compared to 2013, now including Yunnan, Guizhou, Guangxi, Hunan, Guangdong, Jiangxi, Fujian, Zhejiang and Anhui provinces. The mean incidence of Low-Low clusters was 0.14/100,000 in 2014, meaning the incidence of Low-Low clusters in 2014 was higher than in 2013. Incidences in the Hubei Province increased rapidly from 0.08/100,000 in 2013 to 0.34/100,000 in 2014, thus reducing Hubei Province in Low-Low cluster areas in 2014. Cluster maps for 2013 and 2014 demonstrate that the cluster area in China was relatively stable, and that compared to the spatial distribution map of incidence, the Low-Low cluster area in each year tends to be the same as the low incidence area, all of which are located in the south of China.

## Discussion

Exploring the spatial distribution of a disease is beneficial for guiding the development and implementation of disease surveillance and a control plan. The application of ArcGIS10.3 (ESRI, Redlands) in disease surveillance has yielded good results. Based on surveillance data about human brucellosis between 2007 to 2016, the temporal and spatial distribution of incidences in mainland China suggested that brucellosis in mainland China has unique spatial and temporal clustering characteristics.

In recent years, incidences of brucellosis in mainland China have been relatively high and growing rapidly. Incidences in 2014 are about 2.8 times of that 2007. In addition, brucellosis has obvious seasonality. Incidences were higher in spring and summer and reached the highest in May every year. This is largely consistent with findings from related studies [11, 36–38]. The high incidence may be related to the vigorous development of animal husbandry in mainland China in the past 10 years, with a sharp increase in the number of livestock raised, frequent transactions and frequent transfer of livestock. However, grass-roots animal epidemic prevention departments are weak, and existing resources are not enough to meet the current increasing demand for epidemic prevention, making it difficult to deal with prevention and control measures such as immunization, quarantine and treatment of dead animals. High incidence rates of the disease in spring and summer are mainly due to the fact that it is the calving season, meaning that the chances of exposure to placenta increase. At this time, the contact probability of the relevant occupational personnel with abortion products and secretions of the sick livestock is greatly increased. If there are no effective protective measures for these occupational personnel, incidences will continue to increase.

It was found that brucellosis in China tends to show an upward trend from south to north, all of which began to spread from highly affected areas before gradually spreading outward. The gravity-center of brucellosis in China in recent years gradually moved southwest. However, overall migration distance and direction were small, and the gravity-center was always located in Inner Mongolia. The direction of migration was related to the location of the four major pastoral areas in China, which is similar to previously published research results [39]. High incidences of brucellosis were in the north of mainland China, where some areas showed much higher incidences than in the south of China. This may be because the north is the main gathering area of pastoral areas in China, meaning there are greater numbers of livestock being raised there as well as frequent livestock exchange.

A global spatial autocorrelation study demonstrated that brucellosis in China had a positive spatial correlation and clustering between 2013 and 2014, while there was no spatial autocorrelation between 2007 and 2012 or 2015 and 2016. Further local autocorrelation analysis found that the Low-Low concentration areas of brucellosis in China in 2013 were mainly in the southern part of the country, including the provinces of Yunnan, Guizhou, Guangxi, Hunan, Guangdong, Hubei, Jiangxi, Fujian, Zhejiang and Anhui, which is basically consistent with the low incidence areas. In 2014, the Low-Low cluster areas remained in the south of China. Understanding the spatial distribution of brucellosis in China is of great significance for controlling its occurrence and reducing the burden it causes, as well as for optimizing the allocation of resources. A prevention policy of key monitoring should be adopted for High-High cluster areas, while favorable factors for disease control should be found in time for Low-Low cluster areas, so as to provide guidance for infectious disease surveillance. In recent years, incidences of brucellosis in Low-Low areas in China have been clustered in fixed places, meaning the influencing factors of this clustering need further investigation.

At the same time, since brucellosis is a natural epidemic disease, it is not infectious between people, meaning the natural and social environment have a great impact on it. Some natural factors in neighbouring areas may make brucellosis spread more easily, leading to a clustering distribution of the affected areas. It was found that the areas with a high incidence of brucellosis tended to be concentrated in the north of China (Fig. 4). Previous studies [40–42] have found that besides raising livestock, brucellosis may also be connected to indirect environmental conditions such as altitude, rainfall, temperature, wind speed and other climatic factors. Li found that areas with a high incidence also tended to be located at middle and slightly higher altitudes (1600-1800 m), while incidences of brucellosis at other altitudes were relatively low [43]. Low incidence areas tend to be concentrated in the southern part of China. As well as fewer livestock, there may be other influencing factors. These factors require further study.

Our study is not without limitations. Firstly, incidences of human brucellosis were underestimated to some extent, as our data was passively collected by a monitoring system, while surveillance data quality was influenced by comprehensive factors, such as the capacity of local health workers, availability of laboratory diagnostics, levels of awareness about the need to visit doctors and so on, all of which may have affected the study’s accuracy. Secondly, our analysis was based on provincial-level data. Smaller spatial units may provide more location-specific information about the design and implementation stages of public health programs. Thirdly, this study only explored the clustering areas of brucellosis and did not analyze factors affecting the clustering distribution, therefore neglecting the impact of human and natural environment factors. In the future, we will therefore further explore the temporal and spatial distribution of brucellosis as well as the factors affecting its distribution.

In summary, given developments in animal husbandry in China, the prevention and detection of brucellosis urgently need to be strengthened. This spatial clustering study of brucellosis is helpful for identifying high-risk areas for brucellosis, and to a certain extent provides a basis for the decision-making of relevant departments. In terms of the distribution characteristics of brucellosis epidemic in this study, we suggest that the detection of brucellosis in northern China should be further strengthened, and that effective methods for controlling brucellosis should be found in southern China, where incidence of brucellosis is low. Governments at all levels should attach importance to the establishment of joint prevention and control mechanism among high-incidence areas. Relevant departments in different regions should strengthen both prevention and control throughout the year. More resources for prevention and control should be appropriately increased in these areas to curb the spread of brucellosis.

## Conclusion

It may be concluded that human brucellosis continues to be a widespread challenge in mainland China, especially in the northwest provinces, which are high risk areas. This study utilized ArcGIS10.3 (ESRI, Redlands) to analyze the temporal and spatial distribution of brucellosis in China, thus contributing to the study of high incidence seasons and areas for brucellosis. However, further research should focus on an analysis of environmental, humanistic and socio-economic factors in order to determine risk factors affecting the occurrence and transmission of brucellosis. Such information has the potential to provide critical guidelines for policy makers to initiate prevention measures and control strategies, aimed at susceptible areas that might be high risk, and to prevent or lessen the incidence of human brucellosis in these areas.

## Data Availability

The datasets analyzed during the current study are available from the corresponding author on reasonable request. The URL link of the dataset is: http://www.phsciencedata.cn/Share/ky_sjml.jsp?id=aafa8285-42ae-4dbc-a828-152c2cef6396

## Abbreviations

CDC: Centers for Disease Control and Prevention
WHO: World Health Organization
GIS: Geographic Information System
LISA: Local indicators of spatial association
